# Effect of different injection rates and doses of contrast medium on the image quality of computed tomographic angiography in African grey parrots (*Psittacus erithacus*)

**DOI:** 10.1186/s12917-022-03524-w

**Published:** 2022-12-09

**Authors:** Wen-Lin Wang, Fang-Lun Chang, Pin-Huan Yu

**Affiliations:** 1 Institute of Veterinary Clinical Science, School of Veterinary Medicine, National Taiwan University, Number 153, Section 3, Keelung Road, Da’an District, Taipei, 10672 Taiwan; 2 National Taiwan University Veterinary Hospital, Number 153, Section 3, Keelung Road, Da’an District, Taipei, 10672 Taiwan

**Keywords:** African grey parrots, Atherosclerosis, Computed tomography angiography, Contrast medium, Image quality

## Abstract

**Background:**

Atherosclerosis is a common cardiovascular disease in parrots but the *antemortem* diagnosis is challenging. In human medicine, computed tomography angiography (CTA) has been used widely for the diagnosis of atherosclerosis. By adjusting the injection rate and total dose of contrast medium, the image quality can be improved. To test the effects of different injection conditions on the image quality of major arteries, 10 African grey parrots (*Psittacus erithacus*) were used. Three injection rates (0.3, 0.4, 0.5 mL/s) and three doses (740, 370, 222 mg of iodine/bird) were tested while the other variables of the studies were fixed.

**Result:**

A higher injection dose led to a significantly higher attenuation, image noise and diameter, with a lower signal-to-noise ratio and contrast-to-noise ratio of the six major arteries. The 370 mg of iodine/bird group showed significantly better subjective image quality. Furthermore, with increasing injection rates, the prevalence of heterogeneity decreased. However, we found an increased risk of injection failure for the 0.5 mL/s groups.

**Conclusion:**

We recommend a combination of 370 mg of iodine/bird with 0.4 mL/s for clinical use to achieve better image quality for CTA.

## Background

Atherosclerosis is a common finding upon *postmortem* examination in companion *Psittaciformes* [1]. The prevalence of atherosclerosis in parrots has been found to be 1.9%–91.8% on *postmortem* examinations [2]. Among these species, African grey parrots (*Psittacus erithacus*; AGPs) and Hispaniolan Amazon parrots (*Amazona ventralis*) have the highest prevalence.

Diagnosing atherosclerosis *antemortem* in birds is difficult [3–9]. A tentative diagnosis can be made based on clinical presentation and risk factors for atherosclerosis, but a highly presumptive diagnosis might rely primarily on diagnostic imaging [10–12]. Computed tomography angiography (CTA) is a valuable tool and may become a “gold standard” for diagnosing avian atherosclerosis.

CTA guidelines in human medicine (HM) are well-documented [13, 14]. Some factors can affect vessel enhancement and image quality, and can be divided into three categories: patient, CT, and contrast medium (CM) [15]. With different patient conditions or target organs, practitioners can adjust the factors accordingly to enhance image quality. Little work has been done on how these factors influence image conditions in avian patients.

A study of nine AGPs found that it was easier to interpret the blood-vessel diameter post-contrast at the body window [16]. Another study of nine AGPs employed three timings for saline flush during CM infusion and found that saline flush after CM infusion elicited the most reliable measurements of major arteries owing to the lower heterogeneity of contrast distribution [17]. The injection rate (IR) effect on contrast enhancement has been studied in HM. With a fixed total amount of CM, a faster IR can increase the blood-delivery rate and total amount of CM delivered, which enhances blood-vessel attenuation [15]. The problem of heterogeneity can be overcome by increasing the IR of CM.

As for injection doses, in a study of Hispaniolan Amazon parrots, high intra-observer agreement was obtained using 720 mg of iodine/kg [18]. In a study of AGPs, ~ 1682 mg of iodine/kg was used [16]. The latter study achieved optimal image quality with “moderate”-to- “strong” intra-observer agreement and inter-observer agreement, but attenuation might have been too high (> 500 HU [Hounsfield unit]) for left and right brachiocephalic trunks and the ascending aorta (AA). Furthermore, CM may cause some side-effects (e.g., nephrotoxicity, allergic reaction) [19]. Therefore, our aim was to decrease the dose of CM.

We report the effect of different IRs and doses of CM on image quality in avian CTA. Subsequently, the attenuation of major arteries under different conditions of CM was compared. We hypothesized that: (a) with an increased IR of CM, the heterogeneity of CM distribution would be limited and elicit better image quality; (b) with a reduced dose of CM, attenuation of major arteries would decrease.

## Results

### Animal condition and CTA

All AGPs were healthy based on the examinations mentioned above before conducting CTA.

During recovery following an injection protocol of 0.4 mL/s with 222 mg of iodine/bird, mild crackles were detected at the left flank via auscultation in one of 10 AGPs. Two of the 10 AGPs regurgitated during recovery following an injection protocol of 0.5 mL/s with 222 mg of iodine/bird. Other adverse effects were not observed in other CM injection protocols. AGPs recovered uneventfully < 1 h after anesthesia.

Among the 90 studies, there were two imaging failures in the same parrot owing to CM leakage from the intravenous catheter. One was during the injection protocol of 0.5 mL/s with 740 mg of iodine/bird, and the other was during the injection protocol of 0.5 mL/s with 222 mg of iodine/bird.

### Objective (quantitative) image quality

**Fig. 1:**
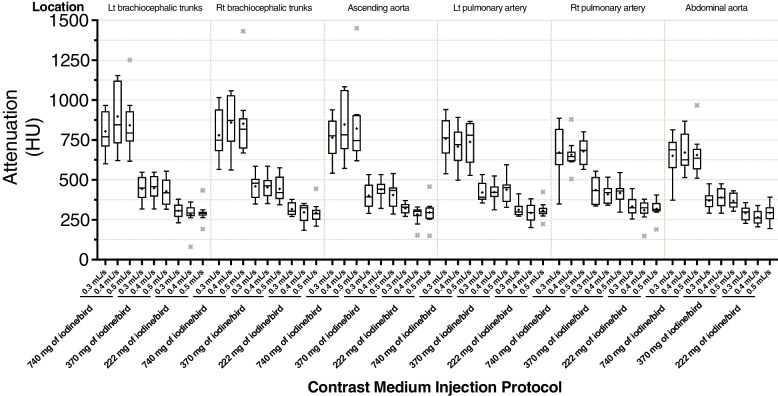
Boxplot of average attenuation (HU) within six major arteries. Vascular enhancement in the groups administered 740 mg of iodine/bird is significantly higher than that of the lowest dose groups. Attenuations with identical doses but different injection rates are nearly identical at all levels. Statistical outliers that are > 1.5-times the interquartile range are represented by ×

A decrease in the CM dose led to a gradual decrease in attenuation (Fig. 1). RM-ANOVA showed that mean attenuations of the six evaluated vessels were significantly different among 3 different injection doses groups (*p* < 0.001). Group of 740 mg of iodine/bird has significantly higher attenuations of six vessels than group of 222 mg of iodine/bird during the post-hoc test (*p* < 0.0006). There was no significant difference between attenuation measured with different IRs.

**Fig. 2:**
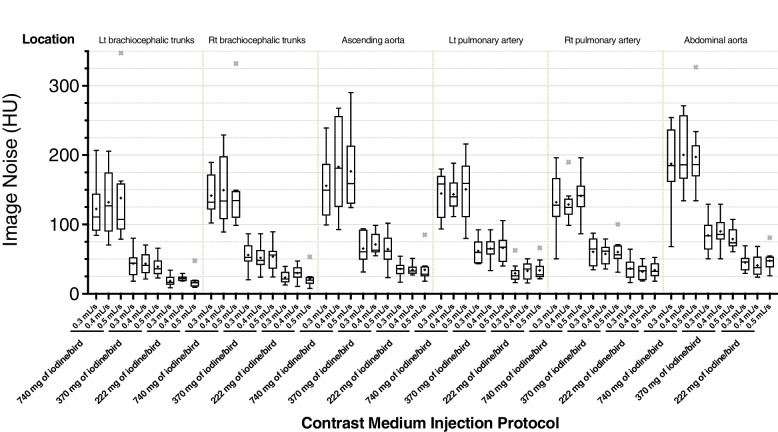
Boxplot of image noise. As the doses of the contrast medium decrease, image noise also gradually decreases. However, with the three injection rates we tested, image noise remained similar. Statistical outliers that are > 1.5-times the interquartile range are represented by ×

A decrease in the CM dose also led to a gradual decrease in image noise (Fig. 2). In the six evaluated vessels, there was a significant and gradual decrease in image noise among 3 groups of different injection doses (*p* < 0.015). All vessels in group of 222 mg of iodine/bird has significantly lower image noise than those in group of 740 mg of iodine/bird (*p* < 0.005).

**Fig. 3:**
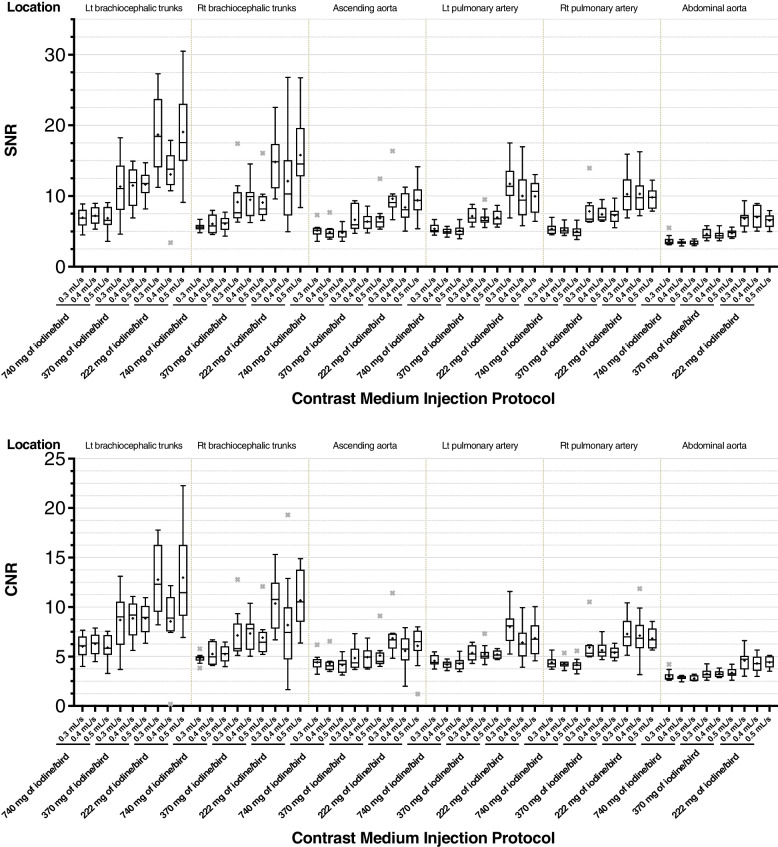
Boxplot of SNR and CNR. A decrease of the contrast-medium dose leads to a gradual improvement in SNR and CNR. However, SNR and CNR were not affected by the three injection rates we tested. Statistical outliers that are > 1.5-times the interquartile range are represented by ×

A decrease in the CM dose led to gradually increases in signal-to-noise ratio (SNR) and contrast-to-noise ratio (CNR) (Fig. 3). SNRs and CNRs of the six evaluated vessels were significantly different among 3 different injection doses groups (*p* < 0.01). The SNRs were significantly higher in the 222 mg of iodine/bird groups than 740 mg of iodine/bird groups (*p* < 0.0277) in most of the vessels except Rt brachiocephalic trunks. Differences in SNR and CNR were not significant among the three IRs tested.

**Fig. 4:**
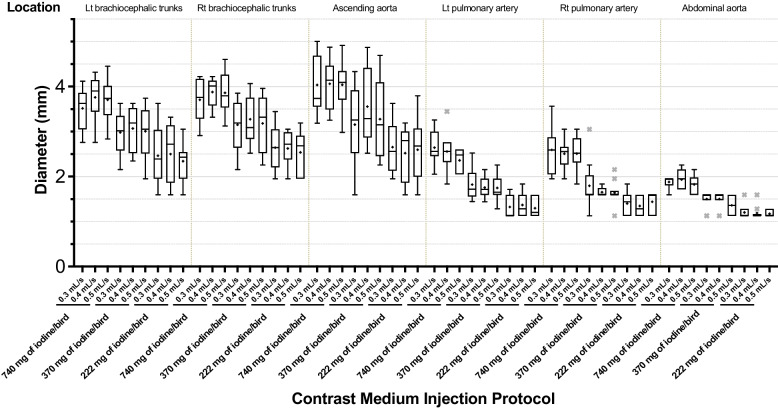
Boxplot of average diameter (mm) of the six measured arteries. The measured vascular diameters are higher in the group of 740 mg of iodine/bird than those in the group of 222 mg of iodine/bird. This phenomenon is more obvious in arteries with wider diameters (i.e., left brachiocephalic trunks, right brachiocephalic trunks, ascending aorta). However, the diameters with the same dose but different injection rates are nearly identical. Statistical outliers that are > 1.5-times the interquartile range are represented by ×

Arterial diameters were significantly larger in the higher dose groups than those of lower dose groups (*p* < 0.001) (Fig. 4). Measured diameters of the six arteries were significantly larger in the group of 740 mg of iodine/bird than the group of 222 mg of iodine/bird (*p* < 0.0011). This phenomenon was more obvious in vessels with wider diameters (i.e., left brachiocephalic trunks, right brachiocephalic trunks, and AA). Arterial diameters were similar among the groups with the same dose but different IRs.

**Fig. 5:**
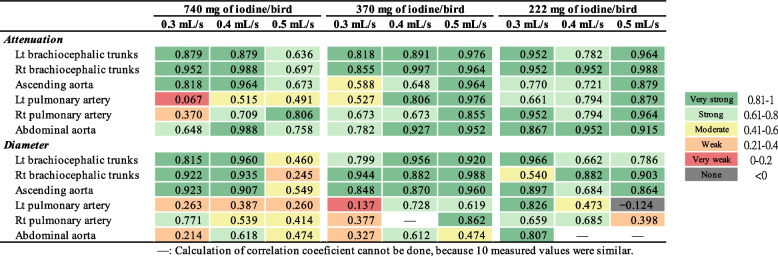
Inter-observer agreement of measured parameters at six major arteries in the nine test groups

**Fig. 6:**
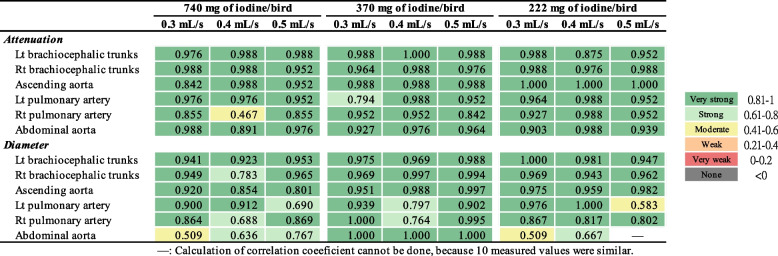
Intra-observer agreement (reproducibility) of measured parameters at six major arteries in the nine test groups

Inter-observer agreements for attenuation and diameter measurement were mostly “strong” to “very strong” in the 370 mg of iodine/bird groups and 222 mg of iodine/bird groups, compared with that in the 740 mg of iodine/bird groups, which has more “weak” to “strong” agreements (Fig. 5). The group with 740 mg of iodine/bird and 0.5 mL/s had the poorest agreements. For intra-observer agreement (reproducibility), in most cases, ICC ≥ 0.8, indicating a “very strong” correlation. Only few exceptions for small arteries (i.e., left and right pulmonary aorta and abdominal aorta) in AGPs undergoing protocols of 740 mg of iodine/bird and 222 mg of iodine/bird had “moderate” agreement (ICC < 0.6) (Fig. 6).

### Subjective (qualitative) image quality

Scoring results for subjective image quality integrated from two observers are listed in Table 1. Subjective image quality score at the six arteries was significantly lower among groups administered 370 mg of iodine/bird *versus* groups given 740 mg of iodine/bird and 222 mg of iodine/bird (*p* < 0.0002) (while score 1 = excellent; 4 = non-diagnostic). Comparison between groups according to different CM doses led to both observers noting a significant degradation of image quality in groups with 222 mg of iodine/bird *versus* other groups. There were more instances of image quality being scored “non-diagnostic” in the 222 mg of iodine/bird with 0.4 mL/s group and 222 mg of iodine/bird with 0.5 mL/s group. The scores of these two groups were significantly higher than those in all the groups with 740 and 370 mg of iodine/bird (*p* < 0.0046).

**Table 1:** Comparison of subjective image quality between nine groups

		**LBCT**				**RBCT**				**Ascending A**				**Left PA**				**Right PA**				**Abdominal A**				**Total [n (%)]**				**Mean score**
	**Score**	**1**	**2**	**3**	**4**	**1**	**2**	**3**	**4**	**1**	**2**	**3**	**4**	**1**	**2**	**3**	**4**	**1**	**2**	**3**	**4**	**1**	**2**	**3**	**4**	**1**	**2**	**3**	**4**	
740 mg of iodine/bird	0.3 mL/s	0	10	0	0	0	10	0	0	0	10	0	0	0	10	0	0	0	10	0	0	0	10	0	0	0 (0%)	60 (100%)	0 (0%)	0 (0%)	2.00 ± 0.00
	0.4 mL/s	0	10	0	0	0	10	0	0	0	10	0	0	1	9	0	0	0	10	0	0	0	10	0	0	1 (1.7%)	59 (98.3%)	0 (0%)	0 (0%)	1.98 ± 0.13
	0.5 mL/s	0	10	0	0	0	10	0	0	0	10	0	0	0	10	0	0	0	10	0	0	0	10	0	0	0 (0%)	60 (100%)	0 (0%)	0 (0%)	2.00 ± 0.00
370 mg of iodine/bird	0.3 mL/s	7	3	0	0	9	1	0	0	9	1	0	0	9	1	0	0	8	1	1	0	9	1	0	0	51 (85%)	8 (13.3%)	1 (1.7%)	0 (0%)	1.17 ± 0.42
	0.4 mL/s	10	0	0	0	9	1	0	0	8	2	0	0	6	2	2	0	5	2	3	0	8	2	0	0	46 (76.7%)	9 (15%)	5 (8.3%)	0 (0%)	1.32 ± 0.62
	0.5 mL/s	6	3	1	0	7	3	0	0	5	4	1	0	8	1	1	0	8	1	1	0	8	2	0	0	42 (70%)	14 (23.3%)	4 (6.7%)	0 (0%)	1.37 ± 0.60
222 mg of iodine/bird	0.3 mL/s	3	2	3	2	4	1	4	1	4	1	4	1	3	0	5	2	2	0	7	1	8	1	0	1	24 (40%)	5 (8.3%)	23 (38.3%)	8 (13.3%)	2.25 ± 1.12
	0.4 mL/s	2	1	2	5	4	1	0	5	2	0	2	6	3	1	2	4	2	0	5	3	7	0	1	2	20 (33.3%)	3 (5%)	12 (20%)	25 (41.7%)	2.70 ± 1.31
	0.5 mL/s	2	1	3	4	3	1	3	3	2	1	2	5	4	1	1	4	3	2	1	4	7	1	0	2	21 (35%)	7 (11.7%)	10 (16.7%)	22 (36.7%)	2.55 ± 1.30

Score classification of image quality: 1 = excellent; 2 = good; 3 = poor; 4 = non-diagnostic

*LBCT* Left brachiocephalic trunk, *RBCT* Right brachiocephalic trunk, *A* Aorta, *PA* Pulmonary aorta

### Time-enhancement curves

Of 90 scans, the time to reach start-scanning cutoff attenuation (100 HU) was similar (< 5 s; which is considered short). A higher frequency of CM heterogeneity was seen in groups with an IR of 0.3 mL/s regardless of CM dose. To quantify CM heterogeneity, subjective scoring for the time-enhancement curve was performed among the nine groups (Table 2). Repeated measurement two-way ANOVA showed that the IR affected the results significantly (*p* = 0.0189): a higher IR led to a lower score, which means lower risk of CM heterogeneity. However, the scores among these groups did not reveal significant difference (*p* > 0.05) during the post-hoc test.

**Table 2:** Comparison of subjective scoring of time-enhancement curves among nine groups

		**Total [n (%)]**			**Mean**
	**Score**	**0**	**1**	**2**	**Score**
740 mg of iodine/bird	0.3 mL/s	1 (10%)	3 (30%)	6 (60%)	1.5
	0.4 mL/s	2 (20%)	5 (50%)	3 (30%)	1.1
	0.5 mL/s	6 (60%)	2 (20%)	2 (20%)	0.6
370 mg of iodine/bird	0.3 mL/s	4 (40%)	2 (20%)	4 (40%)	1.0
	0.4 mL/s	5 (50%)	2 (20%)	3 (30%)	0.8
	0.5 mL/s	6 (60%)	3 (30%)	1 (10%)	0.5
222 mg of iodine/bird	0.3 mL/s	4 (40%)	2 (20%)	4 (40%)	1.0
	0.4 mL/s	6 (60%)	2 (20%)	2 (20%)	0.6
	0.5 mL/s	4 (40%)	6 (60%)	0 (0%)	0.6

Score classification of the time-enhancement curve: 0 = absence of heterogeneity of contrast medium; 1 = mild heterogeneity; 2 = severe heterogeneity

## Discussion

By reducing the CM injection dose, the attenuation of major arteries decreased. By increasing the IR of CM, the heterogeneity of CM distribution was limited, but this did not lead to better image quality.

Attenuation values of the AGPs in our study (600–800 HU) were higher than those of a previous study (300–500 HU) [17] using the same CM protocol (i.e., injection with 2 mL iopamidol followed by 0.4 mL of saline, with an IR of 0.3 mL/s). That result may have been achieved using an optimal delay and precise timing for CT. In our study, scanning was triggered manually once a region of interest (RoI) attenuation of 100 HU was reached; in the previous study, scanning was triggered manually when peak enhancement was reached. The lower attenuation values of the previous study might have been owing to the late scanning-starting time, which may have been too late for timing of highly enhanced attenuation.

**Fig. 7:**
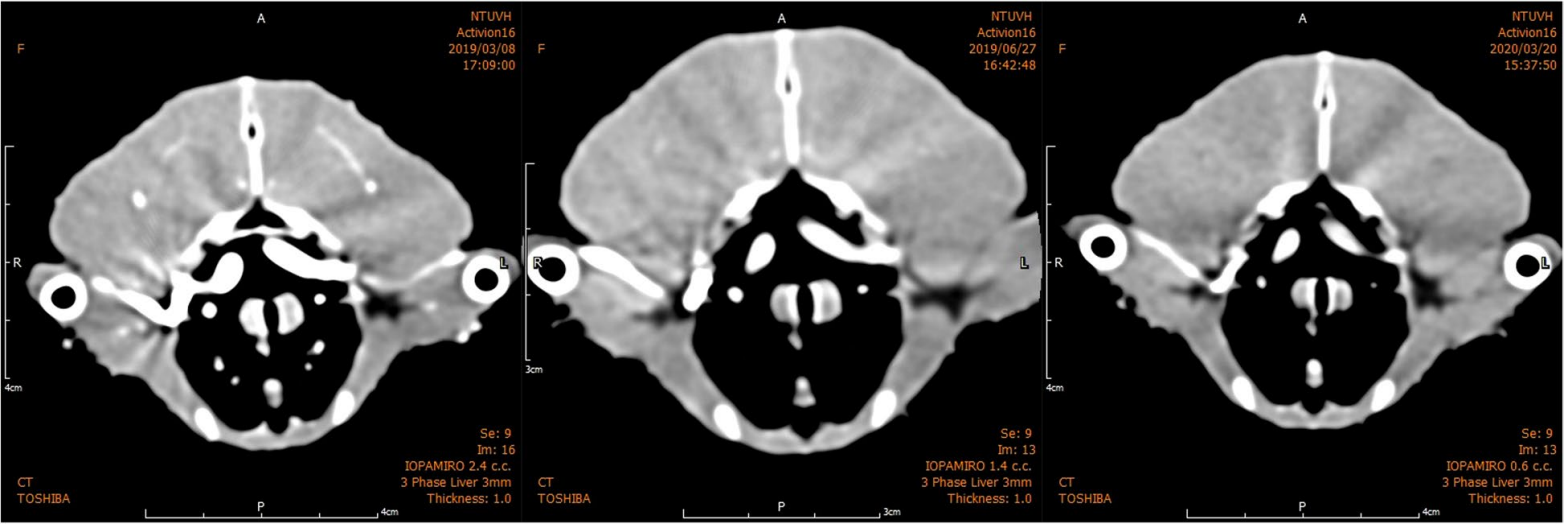
Images under different doses of contrast medium. These three images taken at the same axial plane were acquired from the same bird with different doses of contrast medium. From left to right: 740, 370, 222 mg of iodine/bird. It was obvious that with a higher dosage, the attenuation increases, and the diameter increases due to the blooming artifacts

The minimal enhancement level required for diagnostic CTA for avian arteries is unknown. Diameters of the major arteries of AGPs are similar to those of human coronary arteries (about 2–4 mm) [20], so the regular attenuation of coronary arteries in HM were used as a reference. Optimal attenuation for human vessels is controversial [21]. In humans, atherosclerotic plaques can be calcified or non-calcified. Mean attenuation for calcified plaques is 162–820 HU, and that for non-calcified plaques is 14–125 HU [22]. To distinguish plaques from vessel lumina, optimal vessel attenuation of 250–300 HU has been reported and enhancement > 350 HU may interfere with arterial calcifications [21]. Some scholars postulate that optimal attenuation should be higher for these small arteries and ideally lie at 325–500 HU, and that attenuation > 500 HU is unnecessarily high and leads to an underestimation of coronary calcifications [15, 23–25]. CTA accuracy is affected considerably by vessel attenuation. One study showed that greater attenuation could allow better depiction of vessel lumina, leading to higher diagnostic accuracy [26]. Inadequate attenuation could cause blurring of vessel margins and greater risks of incorrect diagnoses [26, 27]. In the 740 mg of iodine/bird groups, attenuation was too high (> 500 HU). For the 370 mg of iodine/bird groups, attenuation was mostly 325–500 HU. In the 222 mg of iodine/bird groups, attenuation for all the major arteries was about 250–330 HU; nevertheless, the contours of many arteries were blurred and difficult to identify (Fig. 7).

A faster IR is believed to result in higher attenuation compared with that using a lower IR [15]. This phenomenon was not observed in our study, and might have been owing to the short duration of highly enhanced attenuation in AGPs originating from a high cardiac output. The scanning time for these major arteries might start later than the time of peak contrast enhancement, even though whole-image acquisition started immediately after signal attenuation reached 100 HU.

Image noise using a protocol of 740 mg of iodine/bird was significantly higher than that of other study groups. Similar image noise was obtained among different IR groups. In human studies, increased image noise can limit diagnostic accuracy if evaluating small arteries [28, 29] but whether and how image noise affects diagnostic accuracy in birds is unknown. SNR and CNR were significantly higher in the 222 mg of iodine/bird groups compared with those administered 740 mg of iodine/bird. This was because the image noise of the 740 mg of iodine/bird groups was too high, which led to reductions in SNR and CNR.

**Fig. 8:**
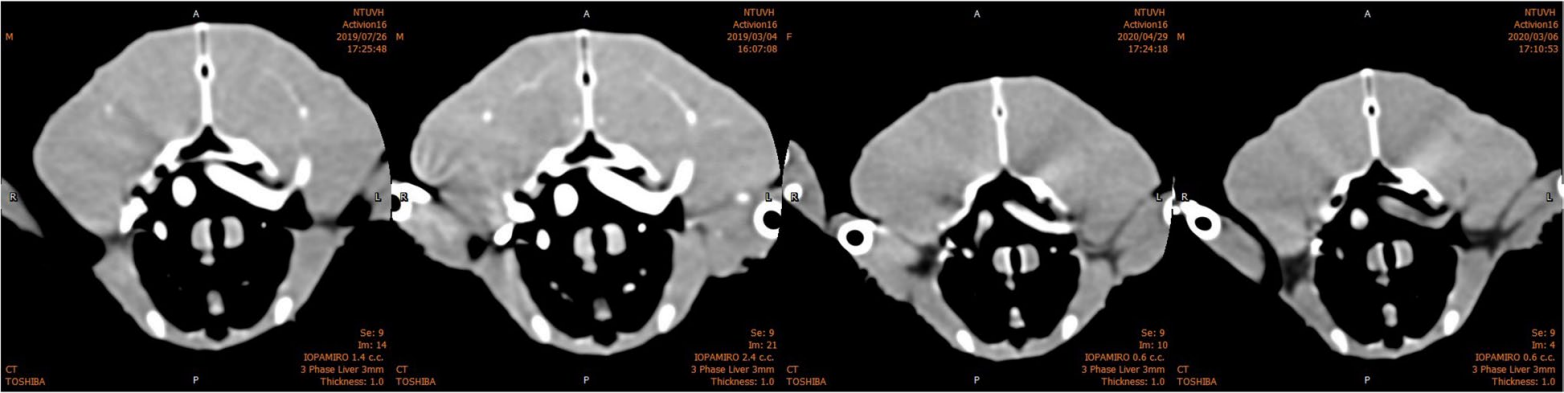
Image examples for each score for the image quality of CTA. From left to right: Score 1 (Excellent), good vessel visualization, vascular contour and lumen are easily detectable; Score 2 (Good), suboptimal attenuation of vessel lumina causing blurring of the contour; Score 3 (Poor), vascular lumen remains acceptable continuity but contours are undetectable; Score 4 (Non-diagnostic), poor enhancement of vessels with disruption of the vessel continuity

The diameter of major arteries was influenced by changes in CM injection dose. This finding could be related to the blooming artifact caused by excessive attenuation of vessels (Fig. 8). The blooming artifact distorts high-attenuation objects to appear larger than actuality, and may be superimposed by additional motion artifacts related to high heart rates [30, 31]. In HM, these high-attenuation objects usually are densely calcified plaques or metallic implants (e.g., coronary stents) [30]. Arterial attenuation in our study using 370 mg and 740 mg of iodine/bird was higher than that of calcified plaques (~ 391 ± 156 HU) [22]; therefore, the diameter of arteries may be overestimated at high doses.

With respect to 740 mg of iodine/bird groups, excessive attenuation of arteries caused streak artifacts in several images, but these artifacts did not affect the vessels excessively. Streak artifacts can occur with the appearance of high-attenuation objects, such as metallic implants or vessels filled with high iodine concentrations [30]. In our study, these artifacts were solved with lower doses of CM; other solutions include using a saline bolus flush or a low concentration of CM [30].

We documented two failures of CM injection owing to CM leakage from the intravenous catheter using an IR of 0.5 mL/s. The maximum IR of CM is influenced by catheter size, total injection volume, or patient size [32]. Higher IRs may result in extravasation of the intravenous CM. An IR of 0.5 mL/s did not exceed the recommended maximum IR for CM using a 24-G catheter (1.0 mL/s in children), but the size of an AGP is far smaller than that of a child. This may have been a reason for the high prevalence (6.7% [2/30]) of injection failure for the 0.5 mL/s groups. Our results revealed the non-necessity of IR > 0.5 mL/s.

Crackles of the respiratory system and regurgitation were noted after three CTA experiments. Crackles might be related to CM. In HM, a high osmolarity of intravenous CM can increase microvascular permeability, causing non-cardiogenic pulmonary edema [33]. Nausea and vomiting are common side-effects reported in humans after receiving CM [34]. However, regurgitation might have been owing to a side-effect either from CM or anesthesia.

Our study had three limitations. First, the sample size was small compared to HM studies. Second, only three injection doses were used, which limited identification of the most appropriate dose for CTA in birds. Furthermore, our study used a one-dose-fits-all approach owing to the limitation of the instrument. Taking bodyweight into consideration, using the bodyweight-based dose in larger avian patients would be rational. Third, we did not record the full time-enhancement curve. Further study is needed to justify the timing and duration of peak enhancement to understand the optimum scan timing. Finally, the total volume of CM changed while we made adjustment to the dose. A study using different CM concentrations allowing to vary the quantity (mg) of contrast without altering the volume (mL) administered would be required to evaluate the potential role of each of these parameters.

## Conclusions

We suggest a dose of 370 mg of iodine/bird provides images of better quality (compared with that using 222 mg of iodine/bird), optimal attenuation, lower image noise, higher SNR, and CNR (compared with 740 mg of iodine/bird). We also suggest an IR of 0.4 mL/s to improve the heterogeneity of contrast enhancement. Therefore, we recommend a combination of 370 mg of iodine/bird with 0.4 mL/s for clinical use if complications and injection failures are taken into account.

## Methods

### Ethical approval of the study protocol

The study protocol was approved (NTU107-EL-00001) by the Institutional Animal Care and Use Committee of National Taiwan University. The number of animals used was minimum determined based on previous reports with similar animal species and study designs. All methods were carried out in accordance with the relevant guidelines and regulations. This study is reported in compliance with ARRIVE guidelines.

Ten adult AGPs (four males and six females; 3–8 y) were obtained from commercial breeders/pet shops. The mean bodyweight was 454.4 ± 50.4 g (range, 361–530.5 g).

AGPs were housed in individual stainless-steel cages (60 × 62 × 87 cm) in a well-ventilated room (4 × 4 × 5 m) at 25–30 °C. Each cage contained environment enrichment toys. An opportunity to exercise and socialize in the room was provided once weekly.

AGPs were offered a commercial pelleted diet (Nutribird P19 Tropical and Nutribird G18 Tropical; Versele-Laga, Deinze, Belgium) with one teaspoon of mixed grains, seeds, and nuts (Vitpower parrot and macaw food; Wanfeng, Taipei, Taiwan) daily and were offered tap water ad libitum from a stainless-steel water bowl. AGPs had lived at the facility for ≥ 2 y before experimentation.

Regular physical examination, blood examination, and whole-body radiographs were obtained. A physical examination involving auscultation of the lungs and heart was performed to ensure each AGP was healthy with clear lung sounds, regular heart rhythm, and no heart murmur. Blood examinations comprised manual complete blood count (packed cell volume, and counts of red blood cells, white blood cells [WBCs] and WBC differential counts), serum biochemistry (aspartate aminotransferase, blood urea nitrogen, calcium, cholesterol, creatine kinase, gamma-glutamyl transferase, glucose, lactate dehydrogenase, phosphorus, total protein, triglycerides, uric acid, and electrolytes) using a VITROS® 350 system (Ortho Clinical Diagnostics, Johnson & Johnson, Melbourne, Australia), and protein electrophoresis (SPIFE® 3000; Helena Laboratories, Beaumont, TX, USA). Radiography (KXO-32 s; Toshiba, Tokyo, Japan) included ventrodorsal and right lateral views.

Additional examinations for cardiological evaluation comprised five-lead electrocardiography (AT-1® Smartprint; Schiller, Baar, Switzerland) [35], echocardiography using a ventral midline approach (EnVisor® HD with S12 UltraBand Sector Xducer; Philips Electronics North America, Andover, MA, USA) [36], and polymerase chain reaction for specific pathogens associated with cardiac diseases (i.e., polyomavirus, *Chlamydia psittaci*, and bornavirus). These examinations and the taking of diagnostic samples were performed under anesthesia. AGPs were included only if they were considered healthy following these examinations [37].

### Study design

Three IRs of CM (0.3 [used previously], 0.4, 0.5 mL/s) combined with three test doses of CM (740 mg of iodine/bird [2 mL, used previously], 370 mg of iodine/bird [1 mL], 222 mg of iodine/bird [0.6 mL]) (Iopamiro 370®; Bracco s.p.a., Milan, Italy) were tested [16, 17]. Infusion of 0.4 ml of 0.9% saline solution was followed immediately after the injection of iopamidol. Therefore, there were nine treatment groups. This study had a prospective, crossover design with a washout period of ≥ 1 month between protocols. If artifacts were present on the time-enhancement curve, or other reasons caused imaging failure, a second scan with that specific protocol was undertaken after a washout period.

### Anesthesia protocol

CT was undertaken with AGPs under general anesthesia. AGPs were fasted for 4–6 h before anesthesia, induced by 5% isoflurane (Attane®; Panion & BF Biotech, Taoyuan, Taiwan) and oxygen (2 L/min), in an induction chamber. Then, each AGP was intubated with a 3.0-mm endotracheal tube (Jorgensen Laboratories, Loveland, CO, USA) and a 24-G catheter (Surflo® 24 G × 3/4"; Terumo, Biñan, Philippines) was placed in the basilic vein.

A veterinary anesthesia-delivery system (ADS 2000®; Engler, Hialeah, FL, USA) was connected and used to maintain anesthesia with 3–5% isoflurane depending on the bird’s depth of anesthesia, which was evaluated by pulse oximetry and a CO_2_ detector (9847 V; Nonin Medical, Plymouth, MN, USA), auscultation, involuntary corneal reflex, and muscle tone. Machine settings were: flow rate, 0.8 L/min; breaths, 17–18 times/min; peak inspiratory pressure (PIP), 7–8 mmHg. Supplemental heat was provided by a heating lamp.

To diminish motion artifacts and enable data acquisition, the anesthesia protocol was adjusted once the contrast study was initiated, as follows: 4–5% isoflurane depending on the anesthesia depth; flow rate, 0.6 L/min; breaths, 2 times/min; PIP, 5 mmHg.

Following completion of diagnostic imaging, the intravenous catheter was removed, and the final physical examination was performed to identify related side-effects. Lactated Ringer’s solution (25 mL, subcutaneous) was administered to reduce the potential adverse renal effects of CM. After the procedures, the overall conditions, appetite, fecal output, responsiveness, and behavior were monitored for the following three days.

### CTA protocol

AGPs were positioned in dorsal recumbency on a V-shaped trough during imaging using a 16-detector row CT (Activion 16; Toshiba, Tokyo, Japan). Whole-body “scout” scans were obtained first (cranial-to-caudal) to map the longitudinal field of view of the helical scan. According to the scout image, the scan range was determined from the last cervical vertebrae to the coxofemoral joint. To locate the AA, pre-contrast helical scans were obtained in slice thickness (section collimation) of 0.5 mm, and section width of 3 mm, from cranial-to-caudal, with a peak electric potential of 120 kVp, electrical current of 50 mA, helical pitch of 1.0, rotation speed of 0.5 s, and table feed of 10 mm/s.

The AA path was determined by reference to unenhanced images. During the contrast study, identical scanning parameters were used, and a dynamic scan was undertaken simultaneously using a circular RoI in the middle portion of the AA to monitor attenuation values in time-enhancement curves. CM was delivered by a dual-head power injector system (OptiVantage®; Guerbet, France). Synchronization between CM administration by the injector system and data acquisition was achieved with a real-time bolus-tracking method, and the scan was triggered manually once enhancement reached 100 HU [16, 17].

Images were evaluated using dedicated DICOM viewer software (UniWeb viewer, EBM technologies, Taipei, Taiwan) on a computer workstation (MD570, Asus) with a calibrated LCD flat screen grayscale monitor (MDC2010-2LC, 20.1 inches, Chilin). Images were analyzed through a body (WW 400; WL 40) window with 512 × 512 screen resolution.

### Objective (quantitative) image quality

Parameters of quantitative image quality, including attenuation, image noise, SNR, and CNR, were measured in six major arteries (middle segment of left brachiocephalic trunks; middle segment of right brachiocephalic trunks; AA root; middle segment of the left pulmonary artery; middle segment of the right pulmonary artery; middle segment of abdominal aorta) for each image. Artery diameters were also measured. Cross-sectional images were magnified to 800% to improve measurement accuracy.

To obtain parameters, a circular RoI was placed in each artery by one observer and was enlarged to include the entire vascular lumen; the RoI size was recorded. Attenuation of the six targeted arteries was obtained as mean HU by placing the RoI in each vessel. “Image noise” was defined as the standard deviation (SD) of attenuation (in HU) in an RoI in targeted arteries. Attenuation of the right rhomboideus superficialis muscle was measured at the level of the artery measured using a RoI of area 0.2 cm^2^. To minimize bias from use of a single measurement, measurements of attenuation of arteries, image noise, and attenuation of the rhomboideus superficialis muscle were repeated three times and averaged. These values were used for calculation of SNR, CNR, and diameter.

SNR was defined as mean attenuation (HU) divided by image noise. In HM, CNR is usually defined as the difference between the mean attenuation (HU) of vessels and perivascular fat or muscle divided by image noise. Owing to a lack of perivascular tissue in birds, we chose the rhomboideus superficialis muscle.

Arterial diameter (D) was calculated manually:

$$D = 2 x\left(\frac{area of RoI}{\pi }\right)1/2$$

Two observers participated in RoI measurements. Observer 1 (WWL) obtained all measurements twice to evaluate intra-observer agreement. Observer 2 (YPH) obtained measurements once to evaluate inter-observer agreement.

### Subjective (qualitative) image quality

Two observers evaluated image quality independently, and were blinded to the protocol. “Image quality” was defined by: vascular enhancement, sharpness of the artery contour, artifacts, and diagnostic ability. On the basis of this definition, each reader graded the image quality of the six major arteries independently on a four-point scale (Table 3). The examples of each scoring grade are shown in Fig. 8. In the case of discordant scores between two observers, the higher score was used.

**Table 3:** Scoring scale for the image quality of CTA: descriptive reference

Score	Descriptions
Score 1 (Excellent)	Excellent image quality. Complete absence of artifacts or blurring, which enables sufficient definition of vessel walls and excellent attenuation of vessel lumina
Score 2 (Good)	Good image quality. Minor artifacts, suboptimal attenuation of vessel lumina, vascular contour, and lumen remain easily detectable despite mild blurring
Score 3 (Poor)	Poor image quality. Moderate artifacts, suboptimal attenuation of vessel lumina, minimal-to-mild vessel discontinuity
Score 4 (Non-diagnostic)	Very poor image quality. Non-diagnostic or unacceptable because of poor enhancement of vessels, vessel contours are undetectable, or contain severe artifacts or blurring

### Time-enhancement curves

The time-enhancement curve of each scan was recorded using a smartphone camera upon CM injection. An observer analyzed 90 time-enhancement curves while blinded to the protocol. CM heterogeneity was evaluated in each curve and a qualitative scale from 0 to 2 (0 = absence of CM heterogeneity; 1 = mild heterogeneity; 2 = severe heterogeneity) was scored.

## Statistical analyses

Statistical analyses were undertaken using SPSS 21.0 (IBM, Armonk, NY, USA), Prism 7.04 (GraphPad, San Diego, CA, USA), and Excel™ 2013 (Microsoft, Redmond, WA, USA). For quantitative variables, normality was assessed by the Shapiro–Wilk test. Categorical variables are expressed as frequencies (percentages).

Two-way repeated measures analysis of variance (RM-ANOVA) was conducted to test for differences of objective and subjective image quality between the nine treatment groups with data that met the assumptions of normality and sphericity, whereas differences were calculated through the Friedman test for data that violated these assumptions. The alpha level for all tests was *p* = 0.05. Post-hoc tests were performed using LSD test.

Agreements between attenuation and diameter were explored using intraclass correlation coefficient (ICC). ICC-values were categorized as follows: < 0—no agreement; 0 to 0.20—very weak agreement; 0.21 to 0.40—weak agreement; 0.41 to 0.60—moderate agreement; 0.61 to 0.80—strong agreement; 0.81 to 1—very strong agreement [38].

## Data Availability

The datasets used and/or analyzed during the current study are available from the corresponding author on reasonable request.

## Abbreviations

AGP: African grey parrot
CM: Contrast medium
CTA: Computed tomography angiography
HM: Human medicine
IR: Injection rate
HU: Hounsfield unit
AA: Ascending aorta
SNR: Signal-to-noise ratio
CNR: Contrast-to-noise ratio
RoI: Region of interest
PIP: Peak inspiratory pressure
SD: Standard deviation
CIs: Confidence intervals
